# The Use of Demographics and Psychographics to Study Product Effects with Nutrient Supplements: Exploratory Multi-Country Data

**DOI:** 10.3390/foods10081918

**Published:** 2021-08-18

**Authors:** Herbert L. Meiselman, Carla Kuesten, Jian Bi

**Affiliations:** 1 Herb Meiselman Training and Consulting, Rockport, MA 01966, USA; 2 Amway, Consumer Product Research, Ada, MI 49355, USA; carla.kuesten@Amway.com; 3 Sensometrics Research and Service, Richmond, VA 23236, USA; bbdjcy@aol.com

**Keywords:** demographics, psychographics, product user, wellbeing, price, sensation seeking, neophobia, resilience, compliance, self-efficacy

## Abstract

Demographics and psychographics are used to study the influence of different consumers on product effects in food development and testing. Demographics have a longer history and are routinely used in most research; psychographics are more recent, raising the question of whether they add to research on food products. The research presented here represents extensive exploratory data that demonstrate that both demographic measures and psychographic measures add to our understanding of consumer’s liking ratings for nutrient supplements. The results are discussed in the context of broader research on a range of food products. In addition, the research reported here was conducted in four different countries, demonstrating many country effects. Finally, tests were conducted with users of the products, lapsed users of the product, and users of other nutrient supplements (non-users), and this led to many differences in product testing. These results further suggest that age and gender are not the only demographic variables to be studied, along with psychographic variables. The psychographic variables should be selected for a particular product category under investigation, as effects of specific psychographic measures vary for product categories. Specific variables do not fit all products for both demographics and psychographics.

## 1. Introduction

Both demographics and psychographics are used to study consumer effects in products. Demographics are defined as “the statistical characteristics of human populations (such as age or income) used especially to identify markets” (Merriam Webster online dictionary). More broadly, demographics studies include variables such as age, gender (male, female), income, family situation (alone, partner, married), work status (working, not working), and other factual consumer characteristics. The history of demographics was traced to the work of Paul Cherington in the 1920s.

Psychographics are “market research or statistics classifying population groups according to psychological variables (such as attitudes, values, or fears)” (Merriam Webster online dictionary). More broadly, psychographics involves study of personality, values, attitudes, interests, and lifestyles. Psychographics are subjective; they are not facts. The use of psychographics was traced to the 1930s and the 1940s and the work of Ernest Dichter [1].

While both demographics and psychographics have been used in consumer and market research for many decades, the use of psychographics has increased for research studies. Thus, looking at the number of research papers mentioning psychographics shows an increase from 2011–2021 on Science Direct, the online site for Elsevier publishing with 2500 journals and 40,000 e-books. However, the number of citations of psychographics remains less than 1% of the citations used for demographics. If one looks more closely at papers involving food products, one observes that most of the psychographics papers are in fields of study other than foods, with only a small number of food studies using psychographics. For example, Betancur et al. [2] noted the surprisingly small number of studies of personality variables and beer, given that alcohol consumption research has significance in such studies. They also note the large number of studies of beer with the demographics of age and gender. Psychographics are even more rare in studies of food products rather than more general studies of food orientation or food categories (vegetarian, green shopping, budget shopping, etc.). However, some categories of products related to eating and drinking had psychographic research, including spices [3], wine [4,5], and nutrient supplements [6]. Some investigators approached consumer segmentation in different ways, such as Moskowitz’s “mind genomics” [7], seeking to identify different mind-sets in consumers.

This paper focused on results of cross-cultural product tests of nutrient supplements, which included demographics and psychographics. The demographics included gender, age, product user status, and culture (country). The psychographics included wellness, sense of well-being, product involvement, price quality consciousness, neophobia, sensation seeking, compliance, general self-efficacy, resilience, and mood. Kuesten and colleagues published papers on these and other psychographic measures used with nutrient supplements [6,8,9]. Other references to psychographic testing are given in the Method section and in the Discussion section below. One purpose of the present paper was to greatly expand the list of psychographic measures used in sensory and consumer research and identify those suitable for a health-related product, in this case, a natural food supplement. See Kuesten and Hu [10] for discussion on dietary and food supplements.

There is one exception to the lack of extensive use of psychographics in research related to food. Researchers and clinicians have extensively used psychographic testing related to pathological or disordered eating and drinking. For example, dietary restraint eating was linked to obesity [11]. In general, clinicians focus on negative psychographics, while product development researchers focus on positive psychographics.

This paper presents consumer studies of nutrient supplement tablets conducted in different countries; this permitted the evaluation of demographic and psychographic measures across cultures as well as within a single country. Each of the consumer studies investigated consumer demographics data and psychographic data along with hedonic, sensory, and affective responses.

Demographic and psychographic effects are presented in this paper with a focus on the influence of both on product effects.

## 2. Method

### 2.1. Overall Approach in the Six Studies

The six studies reported in this paper were all conducted with similar methods (Table 1). All were home use tests (HUT) in which a nutrient supplement tablet was provided to people for use over a period of 5 days. Tablets were provided to current users of the tablet, to lapsed users of the tablet, and to persons using other tablet brands (non-users) as specified in Table 1. Demographic and psychographic data were collected before and after daily tablet use except where noted. Research within each country was carried out by the external suppliers listed in Table 1. Within each country, the research questions were translated into the local language as necessary. Further details on the methods used in these studies of nutrient supplements can be found in the studies by Kuesten and colleagues [6,8,9].

### 2.2. Psychographics Measures 

There are hundreds of psychographic tests available to the consumer researcher; many of these are presented in the Handbook of Marketing Scales 2nd edition by Bearden and Netemeyer [12]. Linton et al. [13] reviewed 99 methods for assessing well-being. In this study, we focused on eight aspects of consumer psychographics plus two affective scales. The psychographic tests were previously developed and tested; we used them either without change or adapted them for use in relation to supplement products for product class and purchase decision involvement scales (see (3) below).

(1) Wellness 

Health refers to the biological state of the body, whereas wellness refers to the individual’s assessment of their well-being. Health is objective, while wellness is subjective. The Arizona Integrative Outcomes Scale (AIOS) scale was used in this study to measure consumer well-being. AIOS [14] is a one-item, visual analogue self-rating scale (VAS) with two alternative forms (one for daily ratings, AIOS-24h, and one for monthly ratings, AIOS-1m). The AIOS-1m form was used in this study. The horizontally displayed VAS was 100 mm in length with the low anchor being, “Worst you have ever been” and the high anchor being, “Best you have ever been”.

Linton et al. [13] reviewed wellness questionnaires and noted that 95 out of 99 reviewed contained multiple items, the largest containing 317 items. In contrast, the Arizona Integrative Outcomes Scale (AIOS) has one item.

(2) Sense of Well-Being

Otto et al. [15] explores measures of wellness and discusses integrative well-being and psychological flourishing. We selected one scale item to measure this construct, namely, “I actively look for products and services that help me live a healthy lifestyle”, hereafter referred to as “seek active”.

(3) Product Involvement

Product involvement is a measure of importance or personal relevance. O’Cass [16] distinguished four types of involvement: product involvement, purchase decision involvement, advertising involvement, and consumption involvement. We selected 2 scales with a total of 5 items from Mittal [17] covering product class involvement and purchase decision involvement.

(4) Price–Quality Consciousness

The psychological study of price is often done within the context of the classic work of Lichenstein, Ridgway, and Netemeyer [18] that described 7 roles of price, 5 negative and 2 positive. The positive roles of price are: (1) price–quality schema, and (2) prestige sensitivity [18]. Price quality schema is defined as “the generalized belief across product categories that the level of price cue is related to quality”. Prestige sensitivity is defined as “favorable perceptions of price cue based on feelings of prominence and status that higher prices signal to other people” The 4 items for price–quality schema were built around the issue, “Generally speaking, the higher the price of the product, the higher the quality”.

(5) General Neophobia

Neophobia is a rejection of things or reluctance to try things which are novel or unknown. It is a trait to dislike anything new and fear novelty. Neophobia is distinguished from rejection of familiar things (fussiness or pickiness). Pliner and Hobden [19] developed scales for measuring food and general neophobia; we used the general neophobia scale (GNS) of 8 items measured on a 1–7 scale. The literature has many research reports on food neophobia, especially in children, but very few references for general neophobia. Marie Damsbo-Svendsen et al. [20] reviewed 13 methods to measure food neophobia. Most methods are aimed at children. Only one cited paper/method also measured GNS [19].

(6) Sensation Seeking

Sensation seeking is viewed as “the need for varied, novel and complex sensations and experiences, and the willingness to take physical and social risks for the sake of such experiences” ([21], p. 10). Sensation seeking is related to whether people are variety seekers or are conservative in their variety choices and whether they seek or avoid risk. Zuckerman developed measures of sensation seeking from very long questionnaires to the frequently used Sensation Seeking Scale (SSV) with 40 items [21,22]. The Arnett scale (Arnett Inventory of Sensation Seeking, AISS) [23] has 20 items. Hoyle et al. [24] noted the need for shorter scales for many measurement situations and developed an 8 item scale (BSSS), which was followed by two shorter scales, the BSS4 and the SS2 (see Stephenson [25]). The SS2 was used in the research.

(7) General Self-Efficacy 

General self-efficacy (GSE) is the belief in one’s competence to tackle novel tasks and to cope with adversity in a broad range of stressful or challenging encounters, as opposed to specific self-efficacy, which is constrained to a particular task at hand. The General Self-Efficacy (GSE) scale is a 10 item psychometric scale that is designed to assess optimistic self-beliefs to cope with a variety of difficult demands in life [26].

(8) Resilience

Vella and Pai [27] described resilience as “positive responses or outcomes in the face of significant risk or adversity”. In their review of resilience, they noted the lack of agreement on definition, conceptualization, and measurement of resilience. Chmitorz et al. [28] noted some of the same issues with defining and measuring resilience. It is not agreed whether resilience is a (temporary) state or a (more permanent) trait. Connor and Davidson [29] developed the 25 item Connor–Davidson Resilience Scale (CD-RISC) to measure resilience, with each item measured on a 5-point scale. An additional item, “Thrive in stressful circumstances/environments” was augmented in some studies.

One scale of mood and affect was also included.

(9) Mood

A mood is a transient feeling that people report and is distinguished from an emotion, which is a response to a specific stimulus. The Profile of Mood States (POMS) [30] provides reliable and valid measures of the intensity of week-long and “right now” fatigue and energy mood states.

## 3. Results

The rating means and the corresponding errors, i.e., variances of the means, were the basis of the statistical tests, including multiple comparisons for the research presented in this paper. Mean significant differences (α = 0.05) are indicated by different lower-case letters; *p*-values and test statistics are not provided. Additional results are presented in the Supplementary Tables.

### 3.1. Overall Hedonic Means by Study

Table 2 shows the mean hedonic ratings for the current existing in-market product tested blind in each of the six studies. There were significant differences between the two US products tested in studies two and five but no differences between the two Japan products tested in studies one and four. The US (current vs. NextGen test supplement) represented significant improvements, thereby influencing overall liking ratings positively. The Japan (current vs. NextGen test supplement) formulation changes were smaller and different from those undertaken in the US, and thus parity overall liking was observed. Over all countries and studies, hedonic overall liking was rated parity and lower for the Japan studies and the first US study compared to the final US, Germany, and Thailand studies, which rated significantly higher.

### 3.2. Demographics

Table 3A–C present the demographic results with hedonic mean scores. Scores are presented for each country study. Male mean hedonic scores were higher, absolutely speaking, in studies one, two, three, four, and six. Males had significantly higher scores in study one (Japan), and females had significantly higher scores in study five (US). There were not many gender differences in product ratings; males rated product A higher in study one (Japan), and females rated product J higher in study five. Overall, gender did not yield a large number of significant differences in hedonic means of tested products.

Age results showed significant differences in mean hedonic scores for different age groups (25–34, 35–44, 45–54) for studies three (Germany), four (Japan), and five (US). The oldest group had the highest mean liking scores in two of those studies, and the youngest group had the lowest scores in all three studies. Age showed significant differences in mean liking score for different products for only some products. In study three, products G and F showed significant differences among age groups with ages 55+ showing the highest score. In study four, product I showed significant differences in liking among age groups, with ages 35–44 showing the highest score, and in study five, product J showed significant differences among age groups, with those 45–54 giving the highest scores. Study scores for the youngest group were not always the lowest scores when comparing age groups for product liking of specific products.

Mean hedonic ratings were also examined based on whether the respondents were product users, lapsed users (no longer using the product), or non-users of the test product, meaning they used another product. All three groups were tested in studies two, three, five, and six but were not studied in studies one or four. Users rated products significantly higher than non-users in all four studies. Users also scored products significantly higher, specifically products C, D, and E in study two, products F and G in study three, products J and K in study five, and products L, M, and N in study six. The scores for lapsed users sometimes were not different from users and were sometimes not different from non-users. Overall, the mean hedonic scores of users were consistently higher in studies of products than the scores of lapsed users or users of other products.

### 3.3. Psychographics

#### 3.3.1. Mean Ratings by Country

Table 4 presents the mean ratings on the psychographic scales by country. The results showed that there were many differences in mean psychographic scores among the four countries. Sometimes, each country was significantly different from the others, such as for AIOS, price quality consciousness, neophobia, and resilience. For most other psychographic scales (seek active, sensation seeking, compliance, and self-efficacy), three countries were different than the other countries, showing large cross-country differences. For one scale, involvement, there were only two groups of psychographic scores different from each other. Germany tended to have the lowest psychographic scores for involvement (tied with US), price quality consciousness, and sensation seeking. Japan had the lowest scores for AIOS, seek active, and resilience, and the US had the lowest scores for involvement (tied with Germany) and neophobia. For compliance, the US rated lowest and Thailand highest with Germany and Japan falling in between. Thailand usually had higher scores on the psychographic measures. In summary, the absolute mean psychographic scores could not be easily compared among countries, because scores tended to vary across countries.

The results for the POMS showed large country differences (Table 5) with almost every country showing a significant difference on every one of the eight scales. Further, for most scales of the POMS tested in different countries with different products, the POMS ratings declined with product usage compared to before product usage (Table 6A,B).

#### 3.3.2. Psychographics by Demographics

Table 7 shows that some psychographic scales differed by gender, including AIOS, seek active, sensation seeking, and compliance. Males had higher scores for AIOS, sensation seeking, and compliance. Age groups had significantly different psychographic scores for AIOS, involvement, price quality consciousness, neophobia, sensation seeking, and resilience. In scales where there were age differences, younger people tended to score higher. Psychographic scores differed by user class for every scale except seek active; lapsed users tended to have fewer high scores, but both users and non-users had some high scores.

#### 3.3.3. Average Liking by Psychographic Group

Psychographic groups were divided into high and low scorers to examine statistically significant differences in mean hedonic overall liking ratings; see Table 8A,B.

AIOS varied across studies and across products; AIOS showed significant differences in mean liking for study three (Germany, product G), where high scorers were greater than low scorers, and for study six (Thailand, product L), where high scorers were also greater than low scorers.

For the seek active scale, high scorers were greater than low scorers for most studies and most products, specifically in the US (D), Germany, (E,F) Japan (H,I), and Thailand (M).

For product class involvement, high scorers were greater than low scorers for almost all studies in all countries except product L in Thailand.

For price quality consciousness, high scorers were greater than low scorers for product studies H (Japan) and J and K (US).

For neophobia, low scorers were greater than high scorers for four product studies in the US (products C, J) and Germany (products F, G). Note the reversal of the usual direction for other psychographics, low>high, rather than the usual high>low.

For sensation seeking, there were no significant differences between high and low scorers.

For compliance, high scorers were greater than low scorers for almost all products in all countries except products H and I in Japan.

For self-efficacy, high scorers were greater than low scorers for study products C, J, K, L (all US tests), and M (Thailand).

For resilience, most products studies showed high scorers greater than low scorers, except product studies A, H, I, and L.

Thus, higher psychographic scale scores correlated with higher mean liking scores for product class involvement, compliance, and resilience; these effects occurred across countries. Some psychographic study results showed this same pattern for AIOS, seek active, price quality consciousness, and self-efficacy. Neophobia showed an opposite effect on liking for some product studies, with low greater than high. Sensation seeking showed no significant psychographic effects.

## 4. Discussion

This paper reported the method and the results of six studies of nutrient supplements in four different countries, both western and eastern. The six studies were all home use tests (HUTs), thus they represent realistic use conditions for these products. The different studies gave us the opportunity to compare results using demographics and psychographics for relatively large samples of local consumers (about 200 in four studies and about 600 in two studies). The main outcome measure used in this study was overall liking (9-point hedonic scale), and we reported the differences in liking captured by the different demographic and psychographic measures. The following discussion aimed to put the results in the larger context of studying foods with a focus on health.

### 4.1. The Role of Gender

Females are usually found to be more interested in healthy food products [31], and more females than males take dietary supplements in the US according to two reports (CRN 2019 data: females 79%; males 74% [32]; Mikulic 2020: females 77%; males 68% [33]). However, we saw no significant gender differences in product liking in this study of nutrient supplements. Further, when the specific psychographic scales which showed the largest number of significant differences in mean product liking were examined for gender differences, we saw no differences for involvement and resilience and only a small (but significant) difference for compliance. Thus, it appears that gender did not play a large role in this specific product test, but researchers should carefully consider whether gender might be significant in their planned product research.

Gender did play an expected role for some of the measurements. For example, males scored higher than females in sensation seeking (Table 6A,B), as expected from previous research (see Cross et al. [34] for a meta-analysis of sensation seeking studies).

### 4.2. Effects of Age

Older people usually score higher on wellness [35], but that was not seen in these data (Table 5). There were clear age effects in the results for price quality consciousness and for sensation seeking, where the oldest groups (54+) scored significantly lower than the younger groups. Sensation seeking was usually found to be highest in young people, declining across ages [36], especially for health-related risks. The study of age was limited in these data because there were no young people (under 25) and no old people (over 65) and very few people aged 55–64. This is often the case where companies are studying people who purchase the most products. Researchers looking for age effects need to be sure to include appropriate numbers of people in different age categories. This is especially true for older consumers where a very broad range of ages is included under the category of elderly. Some research needs the inclusion of very elderly people, perhaps ages 80+ or 85+. Doets and Kremer [35] further warn that, when studying healthy eating in seniors, we need to segment seniors into appropriate groups based on their capabilities and tendencies and not treat all seniors as one group.

### 4.3. Effects of Product Usage

In many commercial studies of food products, testing is done with product users. In the research reported here, three different groups were studied: people who used the same product being tested (users), people who stopped using the same product being tested (lapsed users), and people who used a different nutrient supplement (non-users). Overall, users rated test products significantly higher in liking. Liking scores for lapsed users did not show a consistent trend. Further, psychographic scores differed by user group for every psychographic measure except the “seek active” question. However, there was not a clear pattern of which user group scored highest; sometimes it was the user group and sometimes the non-user group, while it was rarely the lapsed user group.

The definition of what user experience means was discussed without a clear definition [37]. Overall, the meaning of user status for this research might involve familiarity with the product, and it might involve stronger motivation for the test product. In fact, Santoso and Schrepp [37] referred to “loyalty” as a component of user behavior. In a different product category than foods, many software products are created by considering targeted users from different countries. Santoso and Schrepp found significant differences in the rated importance of user experience for many software product categories. However, they noted that the impact of culture was considerably lower than the impact of inter-individual differences between persons of the same culture. In addition, both cultures studied showed similar rankings of the importance of user experience.

The lack of clearly higher scores for one user group in the present study suggested that user group demographic was not affected by psychographic tests in any consistent way. This is probably good news, because it might show the independence of these two measures for this product category with these cultures. However, Santoso and Schrepp noted that product type probably had greater effect on the user experience than culture. Similarly, Jaeger and Giacalone [38] found different results for users and non-users of plant-based products in their positive and negative associations, with users showing more positive associations and non-users showing more negative associations, but these results depended on the product category. These results point to the need to consider user–non-user differences for each product category. This is especially relevant for research seeking new consumers for a product, since new customers might be users or non-users of the product category.

### 4.4. Country Differences

There were large differences in hedonic scores and psychographic scores between countries. This emphasizes the point that it is risky to apply results from one country to another country.

There is a growing tendency for product testing to be conducted in multiple countries rather than one market, as was done previously, and this trend is supported by the large differences in liking and in psychographic scores across these countries. Ares [39] discussed contemporary issues in cross cultural testing, all of which are relevant to the research reported here and to most cross-cultural research with foods. The issues are sampling procedures, conceptual equivalence, linguistic equivalence, data collection procedures, and cultural differences in response style.

It is difficult to use the exact same data collection procedures in multiple countries due to differences in test administrators and differences in local customs. It is sometimes difficult to present the identical product samples due to effects of shipping, storage, use of local water, and other issues. Linguistic differences and response style were discussed in the measurement of liking. For example, Asians tend to use the middle part of liking scales, while Americans tend to use a broader range of the scale [40]. Scholz et al. [41] found that the General Self-Efficacy (GSE) scale is universal and uni-dimensional across 25 countries. However, they did note substantial country differences. Ungar [42] studied resilience in young people across 14 cultures, demonstrating that cultural and contextual factors are important in the specific ways that resilience is shown globally.

### 4.5. Liking and Psychographics 

We observed that higher psychographic scale scores correlated with higher mean liking scores for product class involvement, compliance, and resilience and that these effects occurred across countries. The results for product class involvement were perhaps more obvious, finding that those more interested in dietary health supplements tended to give those products higher ratings. This would be of interest when testing health products in a random group of consumers or product users. People not using health products, including healthy foods, might rate those products lower based on their degree of product involvement. Companies often test products with product users, thus this would not be a problem in that case.

The results for compliance and resilience were less obvious. Perhaps those scoring higher in compliance and resilience were more positive in general, leading to higher hedonic scores. There is literature on happy people being more compliant, including both laboratory studies and reports from the COVID pandemic [43] and research showing resilient people being happier.

### 4.6. Price

Results tended to show differences for price–quality consciousness as a function of demographics. In addition, those higher in price–quality consciousness scored higher in product liking in Japan and in the US. Tsalis [44] found that price–quality schema and prestige sensitivity were not related to intention to purchase suboptimal foods. Tsalis also noted the lack of country differences, although all countries tested were in northern and western Europe. Campbell et al. [45] used a different four item measure of the price–quality relationship in a university study. They found that product involvement, price consciousness, and price–quality relationship contributed to willingness to pay for foods by students. It appears that the results of attitudes toward price did not permit clear conclusions on the role of price attitudes for this product category.

### 4.7. What about Neophobia?

General neophobia did not show large effects in these studies. There were country differences in neophobia, with each of the four countries being significantly different from the others and the US showing the lowest general neophobia and Thailand showing the highest. However, the studies reported here reflect neophobia scores among those familiar with the supplement category and may differ from those who are not category users.

### 4.8. What about Sensation Seeking?

Differences in sensation seeking were not associated with differences in product liking (Table 6A,B). However, the reader should not take this result as a reason to discount sensation seeking for all food studies. First of all, this study used the two item Slater scale, and we do not know what would have been the effect of using long scales of sensation seeking [24,25]. Second, and perhaps more importantly, sensation seeking was shown to be related to consumption of more “risky” products such as alcohol as well as a whole range of other risky behaviors [46].

The study of sensation seeking and spicy foods is interesting. Ludy and Mattes [47] used the BSSS to study spicy foods and sensation seeking within small samples of spicy food users and non-users and found no relationship. Stone and Pangborn [48] also found no relationship for sensation seeking (using SSS-V) and simple sweet and salty taste solutions. Byrnes and Hayes [49,50] conducted several studies on spicy foods and personality variables. Additionally, they reviewed the literature from the late 20th century relating spicy foods and sensation seeking. They suggested that Ludy and Mattes’ failure to find a relationship between sensation seeking and spicy food consumption might have been the sensation seeking method used—similar to our question above about the use of the Slater 2-point scale in this study. They also did not test foods but tried to distinguish spicy food users and non-users. Byrnes and Hayes [49] used Arnett’s 20-item AISS and observed that sensation seeking was significantly related to liking spicy foods, liking spicy Asian meals and spicy BBQ ribs, and the intake frequency of chilis and chili-containing foods. They did not find a relationship between individual sensitivity to capsaicin and liking/disliking. Byrnes and Hayes [50] went on to study gender differences in the relationship of sensation seeking to liking and consumption of spicy foods, only seeing that relationship for females.

Galimov et al. [51] investigated sensation seeking and consumption of energy drinks in German adolescents and reported a positive relationship. Males were more likely to consume and initiate consumption of energy drinks, but Galimov et al. did not report sensation seeking results separately for males and females. Mills et al. [52] reported a positive association of dietary restraint and sensation seeking. Hatch et al. [53], in a study on dietary supplements (DS) with soldiers, concluded those who used DS scored higher for sensation seeking and novelty characteristics than non-users. Multivitamins/multiminerals were among the most frequently consumed DS by soldiers.

The conclusion of the present results and the past results is that, while sensation seeking did not appear to be a significant variable in the present study, it should be included in the study of any product with an element of risk, including caffeinated beverages, alcoholic beverages, highly salted foods, and very spicy foods.

### 4.9. Resilience

Resilience is often studied with well-being or wellness, and the two might have a genetic basis [54]. Resilience research often addresses young people and those working in health professions. Published resilience research in a product context is uncommon.

### 4.10. POMS

Results from the POMS showed significant country differences and significant declines in POMS results following product use. Kuesten and colleagues published previous papers on the POMS in product tests of nutrient supplements [6].

## 5. Conclusions

Exploration of several global research studies conducted for development of nutrient supplements showed significant consumer demographics and psychographics effects on products. Results of these exploratory studies varied depending on the product differences under investigation and the countries tested. While both demographics and psychographics are important for understanding consumer–product interactions, including psychographics in this research provided deeper understanding of the consumer and the user experience beyond demographics alone. Care should be taken to select or develop demographic and psychographic scales that are relevant and useful for the product category under investigation. This might require some pre-testing of research methods. There is the potential for large country differences in psychographics testing, in seeing the effects of different demographics, and in seeing effects of demographics and psychographics on product testing. However, the consumer insights obtained through the combined use of demographics and psychographics may be strategically and tactically applied by marketing, leading to targeted consumer segmentation efforts.

## Figures and Tables

**Table 1:** Methodology table.

Study	1. Japan (2014)	2. US (2015)	3. Germany (2017)	4. Japan (2019)	5. US (2019)	6. Thailand (2019)
Sample size	206	557	182	206	217	673
Males	105	238	77	98	113	313
Females	101	319	105	108	104	360
Age range	25–55	25–55	18–55	25–55	25–55	18–55
25–34 (n)	54	170	48	56	70	306
35–44 (n)	86	155	49	87	77	223
45–54 (n)	66	232	64	63	70	144
55+ (n)	0	0	21	0	0	0
						
Product user *	206	112	44	206	67	344
Non-user **	0	415	129	0	141	317
Lapsed user ***	0	30	9	0	9	12
						
Number of test products	2	3	2	2	2	3
						
Test products	A = Current vs. B = NextGen test supplement V1	C = Current vs. D = NextGen test supplement V1 and E = NextGen test supplement V2	F = Current vs. G = NextGen test supplement V1	H = NextGen test supplement V1 vs. I = NextGen test supplement V3	J = NextGen test supplement V1 vs. K = NextGen test supplement V3	L = Current vs. M = NextGen test supplement V1 vs. N = NextGen test supplement V2
						
Study Design	Sequential- Monadic	Monadic	Sequential- Monadic	Sequential- Monadic	Sequential- Monadic	Monadic
						
External supplier conducting Home Use Test (HUT) research	IPSOS Japan	P&K Research USA	P & K Research USA (with SAM Sensory and Marketing International in Germany)	IPSOS Japan	P&K Research USA	IPSOS PTE Ltd. (Singapore)
Translation service	IPSOS Japan	No translation required	Multilingual Connections, USA	IPSOS Japan	No translation required	IPSOS Thailand and Nouveu Centric Co., Ltd.

* Current consumer of the test supplement. ** Non-user consumes a vitamin/mineral supplement, but not the test supplement. *** Has not consumed the test supplement within past 6 months.

**Table 2:** Overall liking mean ratings (average of Monday and Friday)—current in-market product effects by study across countries.

Study	Year	Country	Product	Description
5	2019	US	6.89 a	J = NextGen test supplement V1
3	2017	Germany	6.85 a	F = Current
6	2019	Thailand	6.83 a	L = Current
2	2015	US	6.05 b	C = Current
4	2019	Japan	5.97 b	H = NextGen test supplement V1
1	2014	Japan	5.73 b	A = Current

Note: Different lower-case letters beside each mean in product column represents a significant difference at α = 0.05.

**Table 3:** (**A**). Studies 1–6: overall liking mean ratings—demographic effects of gender by study. (**B**). Studies 1–6: overall liking mean ratings—demographic effects of age group by study. (**C**). Studies 1–6: overall liking mean ratings—demographic effects of user type by study.

(A)					
Study	Gender	Supplement			
1. Japan (2014)		Over all supplements	A	B	
	M	6.01 a	6.00 a	6.01 a	
	F	5.62 b	5.50 b	5.73 a	
2. US (2015)		Over all supplements	C	D	E
	M	6.50 a	6.25 a	6.74 a	6.56 a
	F	6.26 a	6.08 a	6.45 a	6.25 a
3. Germany (2017)		Over all supplements	F	G	
	M	6.68 a	6.89 a	6.46 a	
	F	6.62 a	6.89 a	6.35 a	
4. Japan (2019)		Over all supplements	H	I	
	M	6.03 a	5.97 a	6.09 a	
	F	5.85 a	5.87 a	5.82 a	
5. US (2019)		Over all supplements	J	K	
	M	6.70 b	6.71 b	6.69 a	
	F	7.11 a	7.14 a	7.09 a	
6. Thailand (2019)		Over all supplements	L	M	N
	M	7.23 a	6.98 a	7.36 a	7.35 a
	F	7.11 a	6.90 a	7.20 a	7.24 a
**(B)**					
**Study**	**Age**	**Supplement**			
1. Japan (2014)		Over all supplements	A	B	
	25–34	5.61 a	5.62 a	5.60 a	
	35–44	5.85 a	5.82 a	5.87 a	
	45–54	5.95 a	5.80 a	6.10 a	
2. US (2015)		Over all supplements	C	D	E
	25–34	6.39 a	6.30 a	6.40 a	6.48 a
	35–44	6.48 a	6.15 a	6.94 a	6.41 a
	45–54	6.26 a	6.06 a	6.46 a	6.28 a
3. Germany (2017)		Over all supplements	F	G	
	25–34	6.40 b	6.75 b	6.05 b	
	35–44	6.57 b	6.69 b	6.45 b	
	45–54	6.61 b	6.95 b	6.27 b	
	55+	7.49 a	7.50 a	7.48 a	
4. Japan (2019)		Over all supplements	H	I	
	25–34	5.68 b	5.69 a	5.68 b	
	35–44	6.06 a	5.95 a	6.16 a	
	45–54	5.98 ab	6.07 a	5.90 ab	
5. US (2019)		Over all supplements	J	K	
	25–34	6.66 b	6.55 b	6.78 a	
	35–44	6.89 ab	6.94 ab	6.83 a	
	45–54	7.14 a	7.25 a	7.04 a	
6. Thailand (2019)		Over all supplements	L	M	N
	25–34	7.24 a	6.98 a	7.26 a	7.49 a
	35–44	7.16 a	6.95 a	7.30 a	7.23 a
	45–54	7.03 a	6.81 a	7.26 a	6.96 a
**(C)**					
**Study**	**User**	**Supplement**			
1. Japan (2014)		Over all supplements	A	B	
	Test Supplement	5.82	5.76	5.87	
	Lapsed				
	Non-User				
2. US (2015)		Over all supplements	C	D	E
	Test Supplement	7.23 a	6.99 a	7.40 a	7.45 a
	Lapsed	6.72 a	6.33 ab	6.86 ab	7.31 a
	Non-User	6.10 b	5.85 b	6.35 b	6.11 b
3. Germany (2017)		Over all supplements	F	G	
	Test Supplement	7.40 a	7.53 a	7.27 a	
	Lapsed	7.31 a	7.56 a	7.06 ab	
	Non-User	6.34 b	6.62 b	6.05 b	
4. Japan (2019)		Over all supplements	H	I	
	Test Supplement	5.93	5.92	5.95	
	Lapsed				
	Non-User				
5. US (2019)		Over all supplements	J	K	
	Test Supplement	7.36 a	7.34 a	7.38 a	
	Lapsed	6.86 ab	6.94 ab	6.78 ab	
	Non-User	6.68 b	6.71 b	6.65 b	
6. Thailand (2019)		Over all supplements	L	M	N
	Test Supplement	7.38 a	7.12 a	7.49 a	7.50 a
	Lapsed	6.75 ab	5.50 ab	7.50 a	7.83 ab
	Non-User	6.96 b	6.80 b	7.02 b	7.05 b

Note: Upper-case letters refer to test supplements. Different lower-case letters beside each mean across rows within each study and demographic represent a significant difference at α = 0.05.

**Table 4:** Psychographics ratings by country.

	Germany	Japan	Thailand	US
Scale	n = 182	n = 412	n = 673	n = 774
AIOS	7.07 c	6.31 d	8.34 a	7.87 b
Seek active	5.84 c	5.68 c	6.42 a	6.21 b
Product Class Involvement	5.29 b	6.07 a	6.00 a	5.26 b
Price Quality Consciousness	3.76 d	3.94 c	4.75 a	4.24 b
Neophobia	2.84 c	3.33 b	3.94 a	2.40 d
Sensation Seeking	2.18 c	2.78 a	2.43 b	2.37 b
Compliance	3.12 bc	3.22 ab	3.33 a	3.10 c
General Self-Efficacy	3.24 c	2.83 b	3.40 a	3.43 a
Resilience	3.90 c	3.59 d	4.19 b	4.33 a

Note: Different lower-case letters beside each mean across countries (rows) represent a significant difference at α = 0.05.

**Table 5:** Profile of mood states (POMS) before and after product consumption.

	Before Product Consumption				After Product Consumption			
	Germany	Japan	Thailand	US	Germany	Japan	Thailand	US
Scale	n = 182	n = 412	n = 673	n = 774	n = 182	n = 412	n = 673	n = 774
Tension-Anxiety (TA)	6.84 b	11.51 a	5.12 c	6.18 b	3.82 b	8.87 a	2.91 c	3.40 b
Depression-Dejection (DD)	6.96 b	9.20 a	5.55 bc	6.16 bc	3.31 b	5.98 a	2.39 b	2.22 b
Anger-Hostility (AH)	7.31 b	8.79 a	8.20 ab	5.07 c	4.01 b	5.85 a	5.39 a	2.09 c
Vigor-Activity (VA)	17.37 c	14.71 d	22.91 a	18.60 b	16.96 b	13.36 c	23.16 a	16.45 b
Figure-Inertia (FI)	7.68 a	8.41 a	2.845 c	4.82 b	4.20 b	6.28 a	1.31 d	2.17 c
Confusion-Bewilderment (CB)	4.95 b	7.71 a	4.51 b	4.47 b	3.56 b	6.14 a	2.53 d	2.96 c
Friendliness (FR)	19.13 c	14.66 d	20.80 a	20.01 b	16.57 b	12.45 d	20.25 a	15.38 c
Total Mood Disturbance (TMD)	16.36 b	30.91 a	3.32 d	8.10 c	1.94 b	19.77 a	−8.63 d	−3.60 c

Note: Different lower-case letters beside each mean across countries (rows) within the product consumption timepoint (before or after) represent a significant difference at α = 0.05.

**Table 6:** (**A**). Profile of mood states (POMS) before vs. after product consumption by product. (**B**). Profile of mood states (POMS) before vs. after product consumption by product.

(A)														
Study	1. Japan (2014)				2. US (2015)						3. Germany (2017)			
Product	A (n = 206)		B (n = 206)		C (n = 204)		D (n = 179)		E (n = 174)		F (n = 182)		G (n = 182)	
Consumption	Before	After	Before	After	Before	After	Before	After	Before	After	Before	After	Before	After
TA	11.94 a	9.14 b	11.94 a	8.56 b	5.63 a	3.08 b	6.35 a	3.60 b	5.87 a	3.52 b	6.84 a	3.94 b	6.84 a	3.70 b
DD	9.15 a	5.70 b	9.15 a	5.51 b	5.70 a	1.46 b	6.46 a	2.52 b	5.60 a	2.67 b	6.96 a	3.52 b	6.96 a	3.10 b
AH	9.01 a	5.90 b	9.01 a	5.36 b	5.03 a	1.63 b	5.23 a	2.46 b	4.20 a	2.24 b	7.31 a	4.12 b	7.31 a	3.89 b
VA	15.12 a	13.21 b	15.12	13.20 b	19.59 a	17.56 b	18.33 a	16.07 b	19.69 a	15.99 b	17.37 a	17.02 a	17.37 a	16.90 a
FI	8.56 a	6.41 b	8.56 a	5.97 b	4.44 a	1.85 b	4.66 a	2.24 b	4.74 a	2.48 b	7.68 a	4.27 b	7.68 a	4.13 b
CB	7.86 a	6.29 b	7.86 a	6.00 b	4.40 a	2.61 b	4.56 a	3.35 b	4.00 a	3.05 b	4.95 a	3.63 b	4.95 a	3.48 b
FR	14.65 a	12.53 b	14.65 a	12.05 b	20.90 a	16.27 b	19.91 a	15.59 b	20.66 a	15.37 b	19.13 a	16.49 b	19.13 a	16.64 b
TMD	31.40 a	20.22 b	31.40 a	18.19 b	5.60 a	−6.92 b	8.94 a	−1.90 b	4.72 a	−2.04 b	16.36 a	2.47 b	16.36 a	1.41 b
**(B)**														
**Study**	**4. Japan (2019)**				**5. US (2019)**				**6. Thailand (2019)**					
**Product**	**H (n = 206)**		**I (n = 206)**		**J (n = 217)**		**K (n = 217)**		**L (n = 217)**		**M (n = 234)**		**N (n = 222)**	
Consumption	Before	After	Before	After	Before	After	Before	After	Before	After	Before	After	Before	After
TA	11.08 a	8.68 b	11.08 a	9.09 b	6.49 a	3.71 b	6.49 a	3.16 b	5.25 a	2.81 b	5.18 a	3.18 b	4.94 a	2.72 b
DD	9.25 a	6.24 b	9.25 a	6.48 b	6.47 a	2.65 b	6.47 a	1.90 b	5.68 a	2.31 b	5.57 a	2.46 b	5.38 a	2.40 b
AH	8.57 a	5.98 b	8.57 a	6.16 b	5.38 a	2.42 b	5.38 a	1.78 b	8.13 a	5.42 b	8.48 a	5.48 b	7.99 a	5.27 b
VA	14.31 a	13.64 a	14.31 a	13.37 a	17.82 a	16.29 b	17.82 a	16.26 b	22.52 a	23.09 a	23.20 a	23.42 a	22.97 a	22.96 a
FI	8.25 a	6.30 b	8.25 a	6.45 b	5.10 a	2.31 b	5.10 a	2.05 b	2.71 a	1.33 b	3.09 a	1.28 b	2.73 a	1.32 b
CB	7.57 a	6.14 b	7.57 a	6.16 b	4.65 a	3.11 b	4.65 a	2.76 b	4.57 a	2.51 b	4.49 a	2.57 b	4.47 a	2.50 b
FR	14.67 a	12.68 b	14.67 a	12.53 b	19.38 a	15.00 b	19.38 a	14.74 b	20.76 a	20.38 a	20.81 a	20.16 a	20.82 a	20.23 a
TMD	30.41 a	19.69 b	30.41 a	20.96 b	10.28 a	−2.10 b	10.28 a	−4.61 b	3.81 a	−8.71 b	3.61 a	−8.44 b	2.54 a	−8.75 b

Note: Upper-case letters refer to test supplements. Different lower-case letters beside each before and after rating within each product represent a significant difference at α = 0.05.

**Table 7:** Psychographics by gender, age group, and user type.

	Gender		Age Group				User Type		
	Male	Female	25–34	35–44	45–54	55+	Test Supplement	Lapsed	Non-User
Scale	n = 944	n = 1097	n = 704	n = 677	n = 639	n = 21	n = 979	n = 60	n = 1002
AIOS	7.77 a	7.53 b	7.83 a	7.57 b	7.52 b	7.00 ab	7.31 b	7.67 ab	7.96 a
Seek Active	6.09 b	6.18 a	6.15 a	6.10 a	6.17 a	6.00 a	6.15 a	6.25 a	6.12 a
Product Class Involvement	5.69 a	5.62 a	5.70 ab	5.65 b	5.59 b	6.15 a	6.11 a	5.43 b	5.21 b
Price Quality Consciousness	4.31 a	4.29 a	4.41 a	4.34 a	4.16 bc	3.64 c	4.17 b	4.35 ab	4.42 a
Neophobia	3.16 a	3.12 a	3.31 a	3.14 ab	2.96 b	2.70 ab	3.20 a	2.83 c	3.09 b
Sensation Seeking	2.62 a	2.32 b	2.60 a	2.44 b	2.34 b	1.81 c	2.65 a	2.37 ab	2.27 b
Compliance	3.22 a	3.16 b	3.22 a	3.19 a	3.14 a	3.35 a	3.35 a	2.90 b	3.05 b
General Self-Efficacy	3.30 a	3.27 a	3.29 a	3.25 a	3.31 a	3.25 a	3.21 b	3.31 ab	3.36 a
Resilience	4.11 a	4.09 a	4.11 ab	4.05 b	4.14 a	3.85 ab	3.99 b	4.14 ab	4.19 a

Note: Different lower-case letters beside each mean across scales (rows) within demographics represent a significant difference at α = 0.05.

**Table 8:** (**A**). Studies 1–3: average overall liking (Mon and Fri) by psychographic group (low and high). (**B**). Studies 4–6: average overall liking (Monday and Friday) by psychographic group (low and high).

(A)														
Study	1. Japan (2014)				2. US (2015)						3. Germany (2017)			
Product	A (n = 206)		B (n = 206)		C (n = 204)		D (n = 179)		E (n = 174)		F (n = 182)		G (n = 182)	
Scorers	Low	High	Low	High	Low	High	Low	High	Low	High	Low	High	Low	High
AIOS	5.67 a	5.87 a	5.75 a	6.03 a	5.87 a	6.28 a	6.28 a	6.75 a	6.16 a	6.50 a	6.75 a	7.08 a	6.14 b	6.74 a
Seek Active	5.63 a	5.87 a	5.76 a	5.97 a	5.97 a	6.29 a	6.28 b	6.85 a	6.34 a	6.41 a	6.45 b	7.13 a	5.81 b	6.73 a
Product Class Involvement	5.49 b	5.97 a	5.58 b	6.11 a	5.76 b	6.50 a	6.15 b	7.10 a	5.80 b	6.97 a	6.39 b	7.32 a	5.73 b	6.97 a
Price Quality Consciousness	5.78 a	5.74 a	5.92 a	5.83 a	5.99 a	6.31 a	6.48 a	6.65 a	6.37 a	6.38 a	6.90 a	6.88 a	6.32 a	6.40 a
Neophobia	5.78 a	5.74 a	5.91 a	5.84 a	6.41 a	5.81 b	6.49 a	6.67 a	6.48 a	6.25 a	7.10 a	6.64 b	6.62 a	6.14 b
Sensation Seeking	5.72 a	5.79 a	5.81 a	5.92 a	6.02 a	6.27 a	6.75 a	6.40 a	6.27 a	6.49 a	6.92 a	6.85 a	6.47 a	6.30 a
Compliance	5.57 b	5.88 a	5.65 b	6.01 a	5.76 b	6.55 a	6.03 b	6.96 a	6.01 b	6.59 a	6.46 b	7.26 a	5.88 b	6.84 a
General Self-Efficacy	5.68 a	5.85 a	5.79 a	5.97 a	5.76 b	6.53 a	6.33 a	6.77 a	6.40 a	6.36 a	6.78 a	7.01 a	6.25 a	6.56 a
Resilience	5.64 a	5.88 a	5.69 b	6.05 a	5.66 b	6.59 a	6.19 b	6.91 a	6.29 b	6.44 a	6.61 b	7.17 a	6.07 b	6.72 a
**(B)**														
**Study**	**4. Japan (2019)**				**5. US (2019)**				**6. Thailand (2019)**					
**Product**	**H (n = 206)**		**I (n = 206)**		**J (n = 217)**		**K (n = 217)**		**L (n = 217)**		**M (n = 234)**		**N (n = 222)**	
**Scorers**	**Low**	**High**	**Low**	**High**	**Low**	**High**	**Low**	**High**	**Low**	**High**	**Low**	**High**	**Low**	**High**
AIOS	5.97 a	5.84 a	5.86 a	6.08 a	6.87 a	6.94 a	6.74 a	6.96 a	6.75 b	7.12 a	7.28 a	7.26 a	7.36 a	7.23 a
Seek Active	5.66 b	6.12 a	5.66 b	6.17 a	6.54 b	7.24 a	6.54 b	7.17 a	6.80 a	7.05 a	6.98 b	7.50 a	7.19 a	7.38 a
Product Class Involvement	5.73 b	6.04 a	5.63 b	6.16 a	6.40 b	7.35 a	6.39 b	7.30 a	6.86 a	7.00 a	6.98 b	7.55 a	7.10 b	7.48 a
Price Quality Consciousness	5.67 b	6.08 a	5.93 a	5.96 a	6.60 b	7.21 a	6.62 b	7.12 a	6.75 a	7.07 a	7.20 a	7.30 a	7.23 a	7.33 a
Neophobia	5.92 a	5.92 a	6.07 a	5.83 a	7.10 a	6.66 b	6.99 a	6.73 a	6.90 a	6.97 a	7.29 a	7.25 a	7.29 a	7.29 a
Sensation Seeking	5.87 a	5.96 a	5.84 a	6.04 a	7.07 a	6.76 a	7.01 a	6.75 a	6.96 a	6.91 a	7.31 a	7.24 a	7.40 a	7.20 a
Compliance	5.86 a	5.94 a	5.81 a	6.02 a	6.48 b	7.24 a	6.40 b	7.23 a	6.72 b	7.10 a	7.09 b	7.44 a	7.17 a	7.41 a
General Self-Efficacy	5.82 a	6.00 a	5.79 a	6.09 a	6.70 b	7.13 a	6.57 b	7.18 a	6.73 b	7.13 a	7.01 b	7.47 a	7.20 a	7.36 a
Resilience	5.77 a	6.08 a	5.81 a	6.11 a	6.69 b	7.11 a	6.65 b	7.08 a	6.83 a	7.03 a	7.03 b	7.48 a	7.18 a	7.37 a

Note: Upper-case letters refer to test supplements. Different lower-case letters beside each low and high rating within each product represent a significant difference at α = 0.05.

## Supplementary Materials

The following are available online at https://www.mdpi.com/article/10.3390/foods10081918/s1, Table S1A. Studies 1–6: overall liking mean ratings - demographic effects of gender by study. Table S1B. Studies 1–6: overall liking mean ratings - demographic effects of age group by study. Table S1C. Studies 1–6: overall liking mean ratings - demographic effects of user type by study. Table S2A. Study 1: average overall liking (Monday and Friday) by psychographic group (low and high). Table S2B. Study 2: average overall liking (Monday and Friday) by psychographic group (low and high). Table S2C. Study 3: average overall liking (Monday and Friday) by psychographic group (low and high). Table S2D. Study 4: average overall liking (Monday and Friday) by psychographic group (low and high). Table S2E. Study 5: average overall liking (Monday and Friday) by psychographic group (low and high). Table S2F. Study 6: average overall liking (Monday and Friday) by psychographic group (low and high).
