# Supplementary material for: The Use of Demographics and Psychographics to Study Product Effects with Nutrient Supplements: Exploratory Multi-Country Data
Source: Foods. 2021 Aug 18;10(8):1918. doi: 10.3390/foods10081918 (PMC8391947; doi:10.3390/foods10081918)
Supplement: Supplementary file 1 [file foods-10-01918-s001.zip › foods-1305325-supplementary-done.pdf]

SUPPLEMENT TABLES

Table S1A. Studies 1-6: Overall Liking mean ratings - demographic effects of gender by study.

**Study**	**Gender**	**Supplement**			
**1. Japan (2014)**		**Overall all supplements**	**A**	**B**	
	**M**	n=105, 6.01a	n=105, 6.00a	n=105, 6.01a	
	**F**	n=101, 5.62b	n=101, 5.50b	n=101, 5.73a	
**2. US (2015)**		**Overall all supplements**	**C**	**D**	**E**
	**M**	n=238, 6.50a	n=93, 6.25a	n=75, 6.74a	n=70, 6.56a
	**F**	n=319, 6.26a	n=111, 6.08a	n=104, 6.45a	n=104, 6.25a
**3. Germany (2017)**		**Overall all supplements**	**F**	**G**	
	**M**	n=77, 6.68a	n=77, 6.89a	n=77, 6.46a	
	**F**	n=105, 6.62a	n=105, 6.89a	n=105, 6.35a	
**4. Japan (2019)**		**Overall all supplements**	**H**	**I**	
	**M**	n=98, 6.03a	n=98, 5.97a	n=98, 6.09a	
	**F**	n=108, 5.85a	n=108, 5.87a	n=108, 5.82a	
**5. US (2019)**		**Overall all supplements**	**J**	**K**	
	**M**	n=113, 6.70b	n=113, 6.71b	n=113, 6.69a	
	**F**	n=104, 7.11a	n=104, 7.14a	n=104, 7.09a	
**6. Thailand (2019)**		**Overall all supplements**	**L**	**M**	**N**
	**M**	n=313, 7.23a	n=101, 6.98a	n=109, 7.36a	n=103, 7.35a
	**F**	n=360, 7.11a	n=116, 6.90a	n=125, 7.20a	n=119, 7.24a

Note: Upper-case letters refer to test supplements. Different lower-case letters beside each mean across rows within each study and demographic represents a significant difference at α=0.05.

Table S1B. Studies 1-6: Overall Liking mean ratings - demographic effects of age group by study.

Study	Age	Supplement			
		**Over all supplements**	**A**	**B**	
**1. Japan (2014)**	**25-34**	n=54, 5.61a	n=54, 5.62a	n=54, 5.60a	
	**35-44**	n=86, 5.85a	n=86, 5.82a	n=86, 5.87a	
	**45-54**	n=66, 5.95a	n=66, 5.80a	n=66, 6.10a	
		**Over all supplements**	**C**	**D**	**E**
**2. US (2015)**	**25-34**	n=170, 6.39a	n=62, 6.30a	n=55, 6.40a	n=53, 6.48a
	**35-44**	n=155, 6.48a	n=59, 6.15a	n=49, 6.94a	n=47, 6.41a
	**45-54**	n=232, 6.26a	n=83, 6.06a	n=75, 6.46a	n=74, 6.28a
		**Over all supplements**	**F**	**G**	
**3. Germany (2017)**	**25-34**	n=48, 6.40b	n=48, 6.75b	n=48, 6.05b	
	**35-44**	n=49, 6.57b	n=49, 6.69b	n=49, 6.45b	
	**45-54**	n=64, 6.61b	n=64, 6.95b	n=64, 6.27b	
	**55+**	n=21, 7.49a	n=21, 7.50a	n=21, 7.48a	
		**Over all supplements**	**H**	**I**	
**4. Japan (2019)**	**25-34**	n=56, 5.68b	n=56, 5.69a	n=56, 5.68b	
	**35-44**	n=87, 6.06a	n=87, 5.95a	n=87, 6.16a	
	**45-54**	n=63, 5.98ab	n=63, 6.07a	n=63, 5.90ab	
		**Over all supplements**	**J**	**K**	
**5. US (2019)**	**25-34**	n=77, 6.66b	n=77, 6.55b	n=77, 6.78a	
	**35-44**	n=70, 6.89ab	n=70, 6.94ab	n=70, 6.83a	
	**45-54**	n=70, 7.14a	n=70, 7.25a	n=70, 7.04a	
		**Over all supplements**	**L**	**M**	**N**
**6. Thailand (2019)**	**25-34**	n=306, 7.24a	n=102, 6.98a	n=103, 7.26a	n=101, 7.49a
	**35-44**	n=223, 7.16a	n=73, 6.95a	n=76, 7.30a	n=74, 7.23a
	**45-54**	n=144, 7.03a	n=42, 6.81a	n=55, 7.26a	n=47, 6.96a

Note: Upper-case letters refer to test supplements. Different lower-case letters beside each mean across rows within each study and demographic represents a significant difference at α=0.05.

Table S1C. Studies 1-6: Overall Liking mean ratings - demographic effects of user type by study.

Study	User	Supplement			
1. Japan (2014)		**Over all supplements**	**A**	**B**	
	**Test supplement**	n=206, 5.82	n=206, 5.76	n=206, 5.87	
	**Lapsed**				
	**Non-User**				
2. US (2015)		**Over all supplements**	**C**	**D**	**E**
	**Test supplement**	n=112, 7.23a	n=49, 6.99a	n=35, 7.40a	n=28, 7.45a
	**Lapsed**	n=30, 6.72a	n=15, 6.33ab	n=7, 6.86ab	n=8, 7.31a
	**Non-User**	n=415, 6.10b	n=140, 5.85b	n=137, 6.35b	n=138, 6.11b
3. Germany (2017)		**Over all supplements**	**F**	**G**	
	**Test supplement**	n=44, 7.40a	n=44, 7.53a	n=44, 7.27a	
	**Lapsed**	n=9, 7.31a	n=9, 7.56a	n=9, 7.06ab	
	**Non-User**	n=129, 6.34b	n=129, 6.62b	n=129, 6.05b	
4. Japan (2019)		**Over all supplements**	**H**	**I**	
	**Test supplement**	n=206, 5.93	n=206, 5.92	n=206, 5.95	
	**Lapsed**				
	**Non-User**				
5. US (2019)		**Over all supplements**	**J**	**K**	
	**Test supplement**	n=67, 7.36a	n=67, 7.34a	n=67, 7.38a	
	**Lapsed**	n=9, 6.86ab	n=9, 6.94ab	n=9, 6.78ab	
	**Non-User**	n=141, 6.68b	n=141, 6.71b	n=141, 6.65b	
6. Thailand (2019)		**Over all supplements**	**L**	**M**	**N**
	**Test supplement**	n=344, 7.38a	n=109, 7.12a	n=122, 7.49a	n=113, 7.50a
	**Lapsed**	n=12, 6.75ab	n=5, 5.50ab	n=4, 7.50a	n=3, 7.83ab
	**Non-User**	n=317, 6.96b	n=103, 6.80b	n=108, 7.02b	n=106, 7.05b

Note: Upper-case letters refer to test supplements. Different lower-case letters beside each mean across rows within each study and demographic represents a significant difference at α=0.05.

Table S2A. Study 1: Average Overall Liking (Monday and Friday) by psychographic group (low and high).

**Study**	**1. Japan (2014)**			
**Product**	**A (n=206)**		**B (n=206)**	
**Scorers**	**Low**	**High**	**Low**	**High**
AIOS	5.67a	5.87a	5.75a	6.03a
Seek active	5.63a	5.87a	5.76a	5.97a
Product Class Involvement	5.49b	5.97a	5.58b	6.11a
Price Quality Consciousness	5.78a	5.74a	5.92a	5.83a
Neophobia	5.78a	5.74a	5.91a	5.84a
Sensation Seeking	5.72a	5.79a	5.81a	5.92a
Compliance	5.57b	5.88a	5.65b	6.01a
General Self-Efficacy	5.68a	5.85a	5.79a	5.97a
Resilience	5.64a	5.88a	5.69b	6.05a

Note: Upper-case letters refer to test supplements. Different lower-case letters beside each low and high rating within each product represents a significant difference at α=0.05.

Table S2B. Study 2: Average Overall Liking (Monday and Friday) by psychographic group (low and high).

**Study**	**2. US (2015)**					
**Product**	**C (n=204)**		**D (n=179)**		**E (n=174)**	
**Scorers**	**Low**	**High**	**Low**	**High**	**Low**	**High**
AIOS	5.87a	6.28a	6.28a	6.75a	6.16a	6.50a
Seek active	5.97a	6.29a	6.28b	6.85a	6.34a	6.41a
Product Class Involvement	5.76b	6.50a	6.15b	7.10a	5.80b	6.97a
Price Quality Consciousness	5.99a	6.31a	6.48a	6.65a	6.37a	6.38a
Neophobia	6.41a	5.81b	6.49a	6.67a	6.48a	6.25a
Sensation Seeking	6.02a	6.27a	6.75a	6.40a	6.27a	6.49a
Compliance	5.76b	6.55a	6.03b	6.96a	6.01b	6.59a
General Self-Efficacy	5.76b	6.53a	6.33a	6.77a	6.40a	6.36a
Resilience	5.66b	6.59a	6.19b	6.91a	6.29b	6.44a

Note: Upper-case letters refer to test supplements. Different lower-case letters beside each low and high rating within each product represents a significant difference at α=0.05.

Table S2C. Study 3: Average Overall Liking (Monday and Friday) by psychographic group (low and high).

**Study**	**3. Germany (2017)**			
**Product**	**F (n=182)**		**G (n=182)**	
**Scorers**	**Low**	**High**	**Low**	**High**
AIOS	6.75a	7.08a	6.14b	6.74a
Seek active	6.45b	7.13a	5.81b	6.73a
Product Class Involvement	6.39b	7.32a	5.73b	6.97a
Price Quality Consciousness	6.90a	6.88a	6.32a	6.40a
Neophobia	7.10a	6.64b	6.62a	6.14b
Sensation Seeking	6.92a	6.85a	6.47a	6.30a
Compliance	6.46b	7.26a	5.88b	6.84a
General Self-Efficacy	6.78a	7.01a	6.25a	6.56a
Resilience	6.61b	7.17a	6.07b	6.72a

Note: Upper-case letters refer to test supplements. Different lower-case letters beside each low and high rating within each product represents a significant difference at α=0.05.

Table S2D. Study 4: Average Overall Liking (Monday and Friday) by psychographic group (low and high).

**Study**	**4. Japan (2019)**			
**Product**	**H (n=206)**		**I (n=206)**	
**Scorers**	**Low**	**High**	**Low**	**High**
AIOS	5.97a	5.84a	5.86a	6.08a
Seek active	5.66b	6.12a	5.66b	6.17a
Product Class Involvement	5.73b	6.04a	5.63b	6.16a
Price Quality Consciousness	5.67b	6.08a	5.93a	5.96a
Neophobia	5.92a	5.92a	6.07a	5.83a
Sensation Seeking	5.87a	5.96a	5.84a	6.04a
Compliance	5.86a	5.94a	5.81a	6.02a
General Self-Efficacy	5.82a	6.00a	5.79a	6.09a
Resilience	5.77a	6.08a	5.81a	6.11a

Note: Upper-case letters refer to test supplements. Different lower-case letters beside each low and high rating within each product represents a significant difference at α=0.05.

Table S2E. Study 5: Average Overall Liking (Monday and Friday) by psychographic group (low and high).

**Study**	**5. US (2019)**			
**Product**	**J (n=217)**		**K (n=217)**	
**Scorers**	**Low**	**High**	**Low**	**High**
AIOS	6.87a	6.94a	6.74a	6.96a
Seek active	6.54b	7.24a	6.54b	7.17a
Product Class Involvement	6.40b	7.35a	6.39b	7.30a
Price Quality Consciousness	6.60b	7.21a	6.62b	7.12a
Neophobia	7.10a	6.66b	6.99a	6.73a
Sensation Seeking	7.07a	6.76a	7.01a	6.75a
Compliance	6.48b	7.24a	6.40b	7.23a
General Self-Efficacy	6.70b	7.13a	6.57b	7.18a
Resilience	6.69b	7.11a	6.65b	7.08a

Note: Upper-case letters refer to test supplements. Different lower-case letters beside each low and high rating within each product represents a significant difference at α=0.05.

Table S2F. Study 6: Average Overall Liking (Monday and Friday) by psychographic group (low and high).

**Study**	**6. Thailand (2019)**					
**Product**	**L (n=217)**		**M (n=234)**		**N (n=222)**	
**Scorers**	**Low**	**High**	**Low**	**High**	**Low**	**High**
AIOS	6.75b	7.12a	7.28a	7.26a	7.36a	7.23a
Seek active	6.80a	7.05a	6.98b	7.50a	7.19a	7.38a
Product Class Involvement	6.86a	7.00a	6.98b	7.55a	7.10b	7.48a
Price Quality Consciousness	6.75a	7.07a	7.20a	7.30a	7.23a	7.33a
Neophobia	6.90a	6.97a	7.29a	7.25a	7.29a	7.29a
Sensation Seeking	6.96a	6.91a	7.31a	7.24a	7.40a	7.20a
Compliance	6.72b	7.10a	7.09b	7.44a	7.17a	7.41a
General Self-Efficacy	6.73b	7.13a	7.01b	7.47a	7.20a	7.36a
Resilience	6.83a	7.03a	7.03b	7.48a	7.18a	7.37a

Note: Upper-case letters refer to test supplements. Different lower-case letters beside each low and high rating within each product represents a significant difference at α=0.05.
